# Survival analyses of different treatment modalities and clinical stage for hypopharyngeal carcinoma

**DOI:** 10.3389/fonc.2023.1109417

**Published:** 2023-03-03

**Authors:** Tian-Yun Lin, Tsung-Lun Lee, Yen-Bin Hsu, Shyh-Kuan Tai, Ling-Wei Wang, Muh-Hwa Yang, Pen-Yuan Chu

**Affiliations:** ^1^ Department of Otolaryngology-Head and Neck Surgery, Taichung Veterans General Hospital, Taichung, Taiwan; ^2^ Department of Otolaryngology-Head and Neck Surgery, Taipei Veterans General Hospital, Taipei, Taiwan; ^3^ Department of Medicine, School of Medicine, National Yang Ming Chiao Tung University, Taipei, Taiwan; ^4^ Division of Radiation Oncology, Department of Oncology, Taipei Veterans General Hospital, Taipei, Taiwan; ^5^ Division of Medical Oncology, Department of Oncology, Taipei Veterans General Hospital, Taipei, Taiwan; ^6^ Institute of Clinical Medicine, School of Medicine, National Yang Ming Chiao Tung University, Taipei, Taiwan

**Keywords:** hypopharyngeal carcinoma, surgery-based therapy, chemoradiotherapy, clinical stage, survival

## Abstract

**Objective:**

We investigated the effects of different treatment modalities and clinical stage for hypopharyngeal carcinoma (HPC) patients.

**Methods:**

Between February 2004 and December 2012, 167 HPC patients were reviewed. We calculated overall survival (OS), progression-free survival (PFS), local failure-free survival (LFFS), regional failure-free survival (RFFS), and distant metastasis failure-free survival (DMFFS) using the Kaplan–Meier method and compared various survival outcomes between definitive chemoradiotherapy (CRT) and surgery-based therapy (SBT).

**Results:**

There were no significant differences in baseline characteristics between SBT (n = 102) and definitive CRT (n = 65) groups. The 5-year rates of OS (59.7% vs. 24.0%, p < 0.0001) and PFS (49.9% vs. 22.6%, p = 0.0002) were significantly better in patients who received SBT than in those who received definitive CRT. The SBT group also obtained better LFFS (p < 0.0001), RFFS (p = 0.0479), and DMFFS (p = 0.0110). We did similar analyses by different T-classification (T1–2, T3, and T4) and found that SBT had better OS (p < 0.0001 and p = 0.0020), PFS (p < 0.0001 and p = 0.0513), LFFS (p = 0.0002 and p = 0.0075), RFFS (p = 0.1949 and p = 0.0826), and DMFFS (p = 0.0248 and p = 0.0436) in the T4 and T1–2 subgroups but similar OS (p = 0.9598), PFS (p = 0.5052), RFFS (p = 0.9648), and DMFFS (p = 0.8239) in T3 patients. Analyses by different overall stages revealed no differences between definitive CRT and SBT for stage III patients but significantly better results for stage IV patients who received SBT.

**Conclusions:**

SBT can obtain significant survival benefits when compared with definitive CRT for the whole cohort of patients. Definitive CRT has similar survival outcomes compared with SBT only for T3 tumors or overall stage III disease.

## 1 Introduction

Squamous cell carcinoma of the head and neck (SCCHN) develops from the mucosal epithelium in the oral cavity, oropharynx, hypopharynx, and larynx. SCCHN is the most common malignancy that arises in the head and neck region. It ranks the sixth most common cancer worldwide, with 890,000 new cases and 450,000 deaths in 2018 (1, 2). Among different subsites of SCCHN, hypopharyngeal carcinoma (HPC) has the worst prognosis. The reported 5-year survival rates for stage III and IV HPC are only 36% and 24%, respectively (3). Because of a relatively low incidence rate, literature regarding treatment outcomes for HPC patients is not common, and prospective clinical trials that focused exclusively on HPC are very rare. Most clinical studies enrolled all SCCHN patients and HPC patients accounted for only a small proportion. In general, there are two different treatment approaches for advanced HPC, either surgery-based therapy or definitive chemoradiotherapy (CRT). So far, no consensus has been established on which treatment modality should be the standard of care. In this study, we reported the long-term treatment results of HPC patients and investigated the survival impacts of different treatment modalities, clinical T-classification, and overall stage as a reference for future therapy.

## 2 Materials and methods

### 2.1 Patient selection

The major inclusion criteria for this retrospective study were patients with previously untreated, biopsy-proven squamous cell carcinoma of the hypopharynx who received curative treatment in the Taipei Veterans General Hospital, Taipei, Taiwan, during the period from February 2004 through December 2012. The staging criterion was according to the 7th edition American Joint Committee on Cancer TNM staging system (4). The exclusion criteria were patients with stage I, initial distant metastasis, non-squamous cell carcinoma histology, synchronous multiple primary cancers, incomplete medical charts, and loss of follow-up. This study was approved by the Institutional Review Board of our hospital, and informed consent was waived because of the retrospective chart review without any intervention and contact with patients.

According to our institutional guideline, we offered organ preservation treatment, either transoral laser microsurgery or definitive (chemo)radiotherapy, to clinical T1 and T2 hypopharyngeal carcinoma patients. For clinical T3 tumors with laryngeal dysfunction, such as airway compromise, vocal fold fixation, or post-cricoid tumor with esophageal inlet or inter-arytenoid invasion, we recommended pharyngectomy with total laryngectomy; for those without laryngeal dysfunction, we applied organ preservation treatment, either transoral laser microsurgery or definitive CRT. For the majority of T4 tumors, pharyngectomy with total laryngectomy was advised for operable ones and otherwise patients underwent definitive CRT. Thanks to the National Health Insurance in Taiwan covering nearly 100% of the population, our patients had similar accessibility to definitive CRT and surgery-based therapy with affordable payment. The decision on which treatment strategy to receive depended on not only tumor factors including surgical risks and our institutional guidelines but also patients’ preferences and performance status.

A total of 167 patients entered the final analysis. **Table 1** demonstrates patient characteristics. There were 161 men and 6 women. Their age ranged from 33 to 91 (median 57) years. The primary origin of most patients arose from the pyriform sinus (n = 148) and the remaining from the posterior hypopharyngeal wall or post-cricoid area (n = 19). The clinical stage distribution revealed 16 patients with stage II, 35 with stage III, and 116 with stage IV. Sixty-five patients received definitive CRT, and 102 patients received surgery-based therapy.

**Table 1:** Patient characteristics.

Characteristics	Treatment modality				p
	Surgery-based (n = 102)		Chemoradiotherapy (n = 65)		
	No	%	No	%	
Age (year), median (95% confidence interval)	57.0 (55.0–60.0)		58.0 (54.1–63.0)		0.7852
Sex					1.0000
Male	98	96.1	63	96.9	
Female	4	3.9	2	3.1	
Primary subsite					0.4239
Pyriform sinus	92	90.2	56	86.2	
Others	10	9.8	9	13.8	
Airway obstruction					0.4329
Yes	3	2.9	4	6.2	
No	99	97.1	61	93.8	
Clinical T-classification					0.6797
T1	3	2.9	3	4.6	
T2	23	22.5	18	27.7	
T3	31	30.4	15	23.1	
T4	45	44.1	29	44.6	
Clinical N-classification					0.0918
N0	31	30.4	19	29.2	
N1	19	18.6	7	10.8	
N2	52	51.0	36	55.4	
N3	0	0	3	4.6	
Overall stage					0.9577
II	10	9.8	6	9.2	
III	22	21.6	13	20.0	
IV	70	68.6	46	70.8	

### 2.2 Definitive chemoradiotherapy treatment

The definitive CRT group consisted of concurrent chemoradiotherapy (CCRT; n = 24), bioradiotherapy (BioRT; n = 7), and induction chemotherapy followed by adjuvant CCRT/BioRT/RT (n = 32/1/1).

The most frequently used regimen of induction chemotherapy was triweekly cisplatin and 5-fluorouracil (PF) before 2009. It was shifted to TPF (taxotere+PF) afterward. The concurrent chemotherapy used in these years was triweekly high-dose cisplatin (80–100 mg/m^2^) or weekly low-dose cisplatin 30–40 mg/m^2^. BioRT represented a loading dose of cetuximab 400 mg/m^2^ followed by weekly 250 mg/m^2^ concurrently with radiotherapy (RT). No adjuvant chemotherapy was applied in these patients.

The RT was administered by intensity-modulated radiotherapy (IMRT) technique with a total dose of 70 Gy/35 fractions to the primary tumor site and metastatic regional nodal area plus a 2–4mm margin *via* a conventional fractionation schedule (a daily 2 Gy, 5 days per week).

### 2.3 Surgery-based treatment

Regarding the surgical treatment techniques for the primary site, we offered radical open surgery including total laryngectomy for cases with difficult endoscopic tumor exposure, arytenoid fixation, cartilage destruction, neck soft tissue invasion, or poor pulmonary function and organ preservation therapy with transoral laser microsurgery for the others. As for the neck, ipsilateral or bilateral neck dissection was advised for all patients. Postoperative adjuvant RT/CCRT/BioRT was applied for those with pathological risk features, and the total dose of RT was 60–66 Gy/30–33 fractions. The regimen of postoperative concurrent chemotherapy was mainly weekly cisplatin 25–30 mg/m^2^ with daily oral tegafur–uracil 200–400 mg.

Surgery-based therapy included surgery alone (n = 26), surgery+adjuvant RT (n = 8), surgery+adjuvant CCRT (n = 55), and surgery+BioRT (n = 13). Among these, five patients also received induction chemotherapy.

### 2.4 Survival analyses

Various survival curves were calculated according to the Kaplan–Meier method. Overall survival (OS) was defined as the date from the first day of curative treatment to death of any cause or the date of the last follow-up visit. Progression-free survival (PFS) was defined as the time from the first day of curative treatment to the time of disease progression or death. Local failure-free survival (LFFS), regional failure-free survival (RFFS), and distant metastasis failure-free survival (DMFFS) were calculated from the first day of curative treatment until the day of the primary, neck, or distant relapse or the date of the last follow-up visit. Survival differences between different subgroups were analyzed using the log-rank test. Patient characteristics and other variables were compared, as follows. The Mann–Whitney test was used for age, the continuous variable, of the two groups. The chi-square test was used for categorical or ordinal variables. Fisher’s exact test was used when a small sample size existed. All statistical tests were two-sided, and a p-value of less than 0.05 is considered statistically significant. Analyses were performed by using MedCalc Statistical Software version 20.014 (MedCalc Software Ltd, Ostend, Belgium).

## 3 Results

### 3.1 Long-term treatment outcome

After a median follow-up of 54.3 months, there were 70 recurrences (41.9%) and 117 deaths (70.1%). Failure site distribution included 12 hypopharynx alone, 11 neck alone, 25 distant metastasis alone, 5 combined hypopharynx and neck, 4 combined hypopharynx and distant failures, 7 combined neck and distant failures, and 6 combined hypopharynx, neck, and distant failure. The 5-year OS, PFS, LFFS, RFFS, and DMFFS were 45.9%, 39.3%, 82.1%, 80.1%, and 71.9% respectively.

The detailed causes of death analysis revealed that 48.7% (57/117) patients died of uncontrolled HPC and 51.3% died of other causes. Secondary malignancy (29/117 = 24.8%) and treatment-induced complications (13/117 = 11.1%) were two major causes of non-HPC deaths, followed by intercurrent diseases (11/117 = 9.4%) and unknown (7/117 = 6.0%). The second primary malignancies consisted of cancers of the esophagus (9), lung (9), head and neck (7), hepatocellular carcinoma (1), urothelial carcinoma (1), lymphoma (1), mucoepidermoid carcinoma (1), and neuroendocrine carcinoma (1).

### 3.2 Comparison of survival outcomes between surgery-based therapy and chemoradiotherapy

**Table 1** showed baseline characteristics between two different treatment approaches. There were no statistically significant differences in terms of age, gender, primary subsite, percentage of severe airway obstruction, T-classification, N-classification, and overall stage between the patients who received surgery-based therapy and definitive CRT.

Thirty-eight of 65 (58.5%) patients in the definitive CRT group and 32 of 102 (31.4%) patients in the surgery-based therapy group developed tumor relapse (p = 0.0006). **Table 2** demonstrated the detailed patterns of failure between the two groups. There were higher rates of local (32.3% vs. 5.9%, p < 0.0001), regional (23.1% vs. 13.7%, p = 0.1210), and distant recurrences (32.3% vs. 20.6%, p = 0.0897) in patients who received definitive CRT compared with those who received surgery-based therapy.

**Table 2:** Patterns of failures.

Characteristics	Treatment modality				p
	Surgery-based (n = 102)		Chemoradiotherapy (n = 65)		
	No	%	No	%	
T alone	2	2.0	10	15.4	
N alone	9	8.8	2	3.1	
M alone	14	13.7	11	16.9	
T+N	0	0	5	7.7	
T+M	2	2.0	2	3.1	
N+M	3	2.9	4	6.2	
T+N+M	2	2.0	4	6.2	
Sum of any failures	32	31.4	38	58.5	0.0006
Total failures in T	6	5.9	21	32.3	<0.0001
Total failures in N	14	13.7	15	23.1	0.1210
Total failures in M	21	20.6	21	32.3	0.0897

T, primary; N, regional; M, distant metastasis.

**Figure 1:**
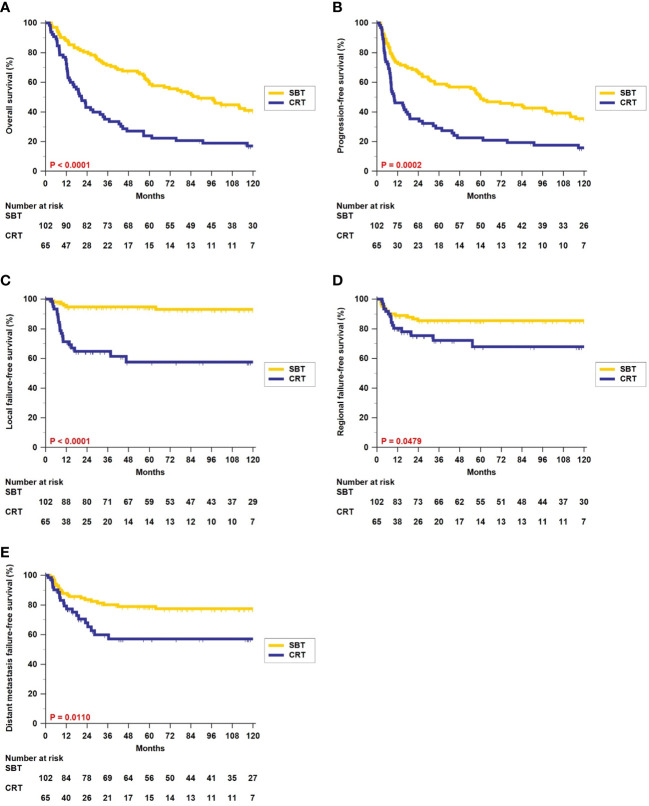
Kaplan–Meier estimates of overall survival **(A)**, progression-free survival **(B)**, local failure-free survival **(C)**, regional failure-free survival **(D)**, and distant metastasis failure-free survival **(E)** between surgery-based therapy (SBT) and definitive chemoradiotherapy (CRT) for all 167 hypopharyngeal carcinoma (HPC) patients.

The 5-year rates of OS (59.7% vs. 24.0%, p < 0.0001, **Figure 1A**) and PFS (49.9% vs. 22.6%, p = 0.0002, **Figure 1B**) were significantly better in patients who received surgery-based therapy than in those who received definitive CRT. Similar results were obtained for the LFFS (94.8% vs. 57.6%, p < 0.0001, **Figure 1C**), RFFS (85.5% vs. 67.9%, p = 0.0479, **Figure 1D**), and DMFFS (79.0% vs. 57.1%, p = 0.0110, **Figure 1E**).

### 3.3 Survival impact by treatment modality in different T-classification subgroups

**Figure 2:**
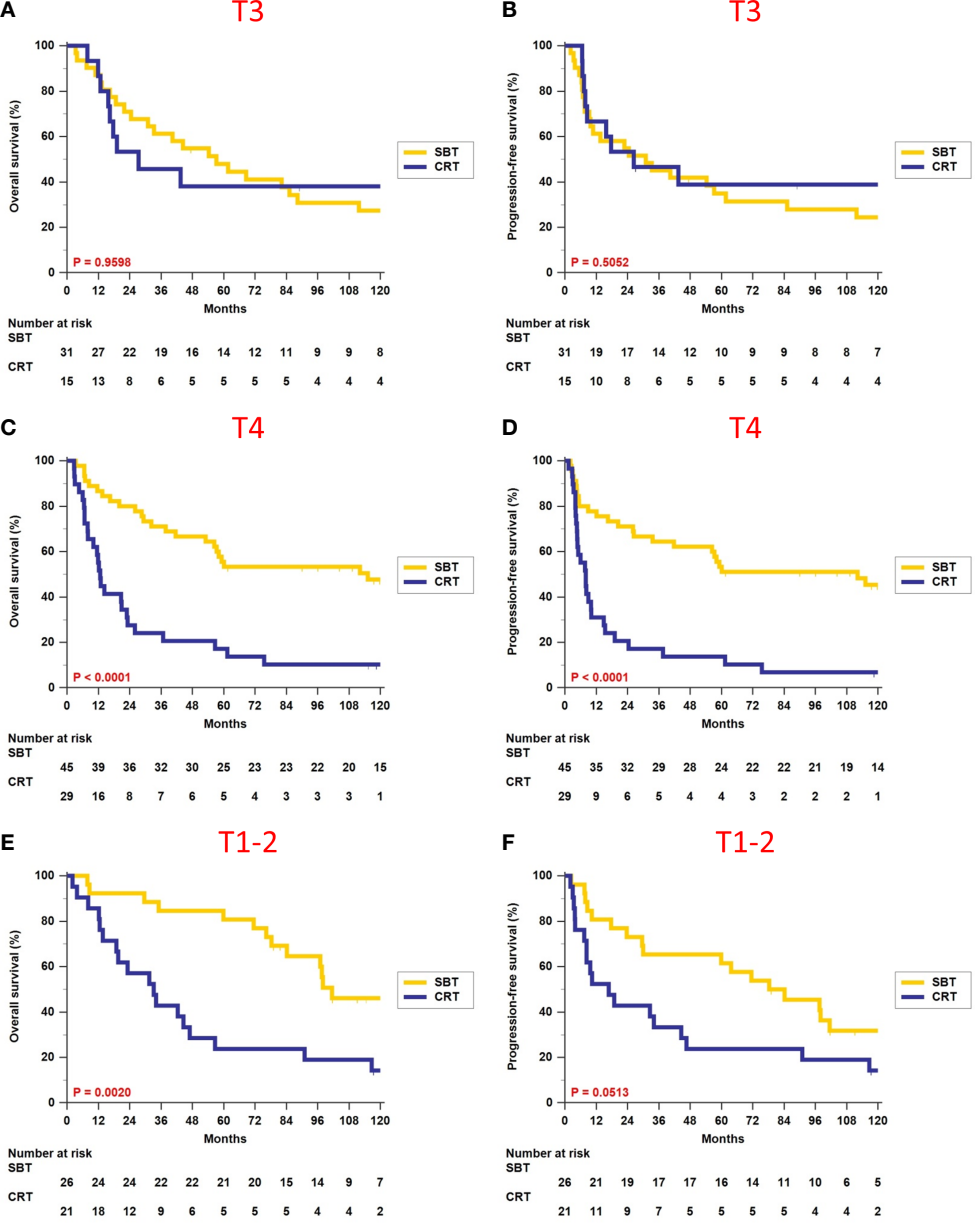
Comparison of overall survival and progression-free survival between surgery-based therapy (SBT) and definitive chemoradiotherapy (CRT) for the T1–2, T3, and T4 subgroups.

We further analyzed the survival impact of surgery-based therapy versus definitive CRT in each different T-classification subgroup. **Table 3** summarizes various survival outcomes between patients who received surgery-based therapy and those who received definitive CRT in the T1–2, T3, and T4 subgroups. Definitive CRT can result in similar OS (5-year rate, 38.1% vs. 48.0%, p = 0.9598, **Figure 2A**) and PFS (5-year rate, 38.9% vs. 34.9%, p = 0.5052, **Figure 2B**) only for the T3 patients compared with surgery-based therapy. The surgery-based approach can obtain significantly better outcomes than definitive CRT in the T4 patients (OS, 5-year rate, 55.6% vs. 17.2%, p < 0.0001, **Figure 2C**; PFS, 5-year rate, 53.3% vs. 13.8%, p < 0.0001, **Figure 2D**) as well as in the T1–2 patients (OS, 5-year rate, 80.8% vs. 23.8%, p = 0.0020, **Figure 2E**; PFS, 5-year rate, 61.5% vs. 23.8%, p = 0.0513, **Figure 2F**).

**Table 3:** Survival comparison between surgery-based treatment versus chemoradiotherapy by T-classification and overall stage.

	Surgery-based vs. chemoradiotherapy				
	OS	PFS	LFFS	RFFS	DMFFS
T1–2 (n = 47)					
5-y rate (%)	80.8 vs. 23.8	61.5 vs. 23.8	95.8 vs. 57.8	87.8 vs. 64.8	91.8 vs. 61.9
p-Value	0.0020	0.0513	0.0075	0.0826	0.0436
T3 (n = 46)					
5-y rate (%)	48.0 vs. 38.1	34.9 vs. 38.9	100.0 vs. 80.0	86.3 vs. 84.8	75.0 vs. 68.8
p-Value	0.9598	0.5052	0.0126	0.9648	0.8239
T4 (n = 74)					
5-y rate (%)	55.6 vs. 17.2	53.3 vs. 13.8	90.7 vs. 42.5	84.0 vs. 61.0	74.3 vs. 46.9
p-Value	<0.0001	<0.0001	0.0002	0.1949	0.0248
Stage II (n = 16)					
5-y rate (%)	90.0 vs. 50.0	70.0 vs. 50.0	100.0 vs. 66.7	80.0 vs. 80.0	100.0 vs. 80.0
p-Value	0.0446	0.2495	0.1943	0.9982	0.5151
Stage III (n = 35)					
5-y rate (%)	58.4 vs. 30.8	40.0 vs. 30.8	95.0 vs. 74.6	85.6 vs. 83.9	85.2 vs. 53.7
p-Value	0.5388	0.8860	0.0942	0.9094	0.0853
Stage IV (n = 116)					
5-y rate (%)	55.7 vs. 18.5	50.0 vs. 16.5	93.9 vs. 49.6	86.7 vs. 61.0	74.1 vs. 54.8
p-Value	<0.0001	<0.0001	<0.0001	0.0273	0.0613

OS, overall survival; PFS, progression-free survival; LFFS, local failure-free survival; RFFS, regional failure-free survival; DMFFS, distant metastasis failure-free survival; 5-y, 5-year.

Regarding patients who received definitive CRT, we noticed that the 5-year OS for the T1–2 subgroup appeared worse than T3, and this trend was consistent among all the other endpoints, namely, PFS, LFFS, RFFS, and DMFFS. After examining the baseline characteristics of these two subgroups, we found that the main difference was clinical N-classification. The T1–2 subgroup contained 33.3% (7/21) N0–1 patients and 66.7% (14/21) N2–3 patients, while the T3 subgroup contained 53.3% (8/15) N0–1 patients and 46.7% (7/15) N2–3 patients. When evaluating their disease severity using overall stage, the T1–2 subgroup had more advanced disease than the T3 subgroup (57.1% vs. 40.0% stage IV disease). The above and the small sample size in these two subgroups may explain why our T1–2 patients had a relatively lower 5-year OS than the T3 subgroup.

### 3.4 Survival impact by treatment modality in different overall stage subgroups

**Figure 3:**
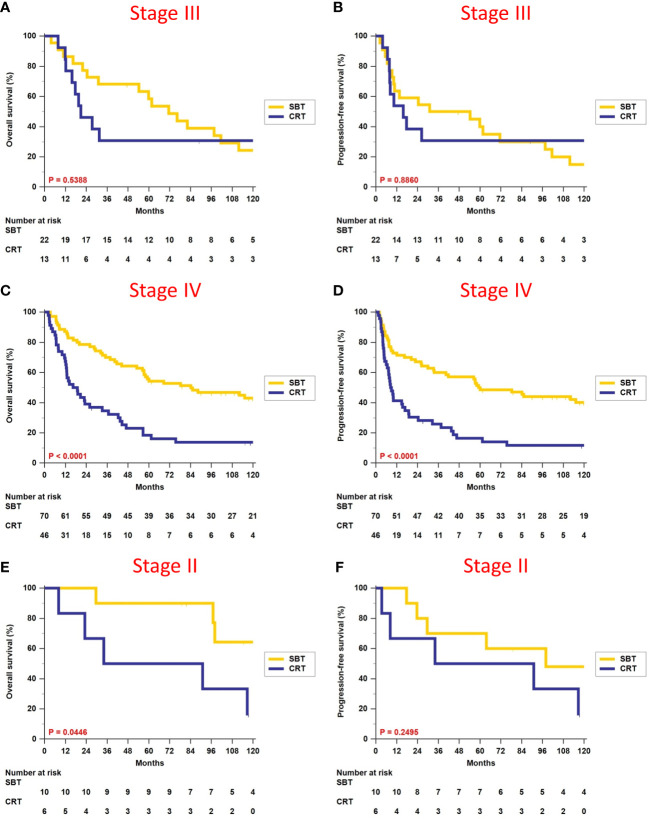
Comparison of overall survival and progression-free survival between surgery-based therapy (SBT) and definitive chemoradiotherapy (CRT) for the stage II, III, and IV subgroups.

We divided patients according to different overall stages II, III, and IV, and did survival analyses between patients who received surgery-based therapy and those who received definitive CRT (**Table 3**). There were no differences in terms of OS (5-year rate, 58.4% vs. 30.8%, p = 0.5388, **Figure 3A**) and PFS (5-year rate, 40.0% vs. 30.8%, p = 0.8860, **Figure 3B**) for patients with stage III disease. Surgery-based therapy had significantly better OS (5-year rate, 55.7% vs. 18.5%, p < 0.0001, **Figure 3C**) and PFS (5-year rate, 50.0% vs. 16.5%, p < 0.0001, **Figure 3D**) benefits than definitive CRT in the stage IV patients. For patients with stage II disease, surgery-based therapy revealed significantly better OS (5-year rate, 90.0% vs. 50.0%, p = 0.0446, **Figure 3E**) and higher rates of PFS at 5 years (70.0% vs. 50.0%, p = 0.2495, **Figure 3F**).

### 3.5 Comparison of survival outcomes between different responses to induction chemotherapy in definitive CRT group

In the definitive CRT group, 34 patients (34/65 = 52.3%) received induction chemotherapy. We recorded their response to induction chemotherapy using the Response Evaluation Criteria in Solid Tumors Group criteria version 1.1 and divided these patients into two subgroups: complete/partial response vs. stable/progressive disease. The 5-year OS, PFS, LFFS, RFFS, and DMFFS were 38.9% vs. 22.5% (p = 0.2026), 38.9% vs. 16.7% (p = 0.0624), 68.1% vs. 27.8% (p = 0.0530), 82.2% vs. 67.1% (p = 0.2984), and 74.3% vs. 50.8% (p = 0.2669), respectively. The complete/partial response subgroup tended to yield better survival outcomes, but the difference did not reach statistical significance.

## 4 Discussion

Treatment strategies for HPC have changed over the past few decades. The traditional treatment approach for locally advanced HPC has been pharyngo-laryngectomy with adjuvant RT ± chemotherapy (5). However, radical surgery will compromise speech and swallowing functions and accordingly result in poor quality of life (6). In 1991, the Veterans Administration Laryngeal Cancer Study Group (VALCSG) published a randomized trial investigating the role of induction chemotherapy (IndCT) with a PF regimen followed by RT in order to spare patients a total laryngectomy (7). This study recruited patients with advanced laryngeal cancer only and showed that 64% of patients in the IndCT arm were able to preserve their larynx and avoid surgical resection. In addition, the 2-year OS rates were the same (68%) for both IndCT and surgical arms (p = 0.9846). The IndCT arm had fewer distant failures (p = 0.016) but more local recurrences (p = 0.0005) and no difference in the overall recurrence rate. Results of the VALCSG trial established the role of IndCT followed by RT and the concept of laryngeal preservation in the treatment of advanced laryngeal cancer. A phase III randomized trial (EORTC-24891) enrolled 202 HPC patients and reported no survival difference between IndCT+RT and surgery+adjuvant RT (8). Long-term follow-up of the EORTC-24891 trial confirmed that the initial larynx-preservation strategy did not compromise disease control or survival (9). Since the publication of both trials, the larynx-preservation approach has become one of the standard treatments for both HPC and laryngeal cancer in clinical practice for approximately three decades.

Many researchers further investigated different larynx-preservation protocols other than IndCT with a PF regimen followed by RT alone. The RTOG-9101 trial allocated patients with laryngeal cancer into three organ-preservation arms, IndCT followed by RT, CCRT, or RT alone, and they found that both chemotherapy-containing arms had significantly better larynx-preservation and locoregional control rates (10). The 2-year rates of larynx-preservation and locoregional control for CCRT vs. IndCT+RT vs. RT alone were 87% vs. 75% vs. 70%, and 78% vs. 61% vs. 52%. However, the OS rates had no significant difference. In the EORTC-24954 trial, Levebvre et al. designed a new schedule of alternating chemoradiotherapy versus the standard IndCT of PF followed by RT for both laryngeal cancer and HPC patients. They observed similar larynx preservation, PFS, and OS, as well as acute and late toxic effects (11). Except for studies focusing on the optimal sequence of combined chemoradiotherapy, a more effective chemotherapy regimen has been explored. In 2007, Vermorken et al. (12) and Posner et al. (13) concomitantly published two important phase III randomized trials (TAX-323 and TAX-324) and showed a higher efficacy of TPF (taxotere plus PF) regimen than conventional PF in all SCCHN. Since then, the TPF regimen has become a preferred IndCT regimen for SCCHN, including HPC in clinical practice. Pointreau et al. reanalyzed data from the TAX-324 trial by selecting patients with HPC and laryngeal cancer only and reported better 3-year rates of larynx preservation (70.3% vs. 57.5%, p = 0.03) and disease-free survival (58% vs. 44%, p = 0.11), favoring the TPF arm (14). However, the OS was similar (60% vs. 60%, p = 0.57). Now, larynx-preservation strategy with definitive CRT (either CCRT alone, IndCT followed by RT/CCRT or alternating chemoradiotherapy) has become more popular than surgery-based therapy in patients with HPC and laryngeal cancer for more than 20 years.

The best treatment strategy for advanced HPC is surgery first or organ preservation approach? It is still a matter of discussion to this day. To the best of our knowledge, there were only three prospective randomized trials focusing on HPC patients (8, 9, 15, 16). As mentioned above, the EORTC-24891 trial demonstrated similar outcomes between surgery-based therapy and definitive CRT (8, 9). The second randomized trial recruited a relatively small sample size (n = 92) and compared two different interventions, IndCT+RT vs. IndCT+total laryngectomy+RT (15). Obviously, this is an unbalance study design, three-combination therapy (chemotherapy, surgery, and RT) vs. two-combination therapy (chemotherapy and RT). The author reported a significantly better OS and local control favoring the three-combination therapy. However, the three-combination therapy arm consisted of total laryngectomy, not meeting the expectation of organ preservation by most patients. The third randomized trial enrolled 71 patients with T3M0 HPC and compared two different organ preservation strategies, CCRT vs. IndCT+RT (16). Results showed no differences in terms of OS, event-free survival, and local control, but CCRT had a higher 2-year larynx-preservation rate (92% vs. 68%, p = 0.016) than IndCT+RT. Based on these limited prospective randomized trials, we could not make a solid conclusion regarding “which treatment approach is better” and may say similar efficacy between primary surgery-based therapy and definitive CRT for HPC patients.

There were several database studies to compare treatment outcomes between surgery-based therapy and definitive CRT. Using the SEER database, Kim and Lee selected 858 HPC patients treated during 2010–2015 and reported similar OS between surgery-based therapy and definitive CRT (17). In contrast, Hochfelder et al. extracted 2,328 HPC patients from the SEER database treated during 2004–2015 and showed better OS and disease-specific survival, favoring the surgery-based therapy arm (18). Another study by Hochfelder et al. enrolled 6055 HPC patients from the National Cancer Database (NCDB) treated during 2004–2015 and found better OS (p < 0.0001) in the surgery-based therapy compared with definitive CRT (19). Kuo et al. analyzed 16,248 HPC patients treated during 1998–2011 from the NCDB and showed that 5-year OS rates were higher for chemoradiotherapy compared with RT alone in the definitive setting but were comparable between “surgery with chemoradiotherapy” and “surgery with RT”. They did not compare outcomes between surgery-based therapy and definitive CRT (20). Multivariate Cox survival regression analysis focused on 3,357 HPC cases diagnosed between 2003 and 2006 without missing data revealed that surgery with RT (HR = 0.772; p = 0.028) and surgery with chemoradiotherapy (HR = 0.693; p = 0.005) were found to be associated with improved survival compared with chemoradiotherapy (20). Two studies from Taiwan revealed that surgery-based therapy had a better outcome than definitive CRT (21, 22). Using data of 2,196 HPC patients derived from the Taiwan Cancer Registry Database and the Taiwan National Health Insurance Research Database between the years 2004 and 2014, and the propensity-score method, Machuca et al. found that surgery-based therapy had better OS (p < 0.0001) (21). Tsai et al. studied 652 HPC patients from the Cancer Registry and Death Registration of Chang Gung Medical Foundation (four Chang-Gung Memorial Hospitals at Linkou, Kaohsiung, Chiayi, and Keelung) and also illustrated better OS (p < 0.001) and disease-free survival (p = 0.003) for the surgery-based therapy arm (22). Based on the above database studies, we may conclude that surgery-based therapy is better than definitive CRT for HPC patients.

Because of the inconsistent results of limited prospective randomized trials mentioned above and database studies, we further did a literature review and selected several observational studies with relatively large patient numbers (**Table 4**) (23–31). Three studies showed significant survival benefits favoring surgery-based therapy. Another six studies reported no significant difference between surgery-based therapy and definitive CRT. Results of the current study showed significantly better survival outcomes favoring surgery-based therapy compared with definitive CRT for all 167 patients. However, subgroup analyses obtained an important but rarely reported point of view—a definitive CRT approach can achieve the same survival outcome as surgery-based therapy only for patients with T3 tumor or overall stage III disease. This new argument may apply to the shared decision-making with the patients in our daily practice. When counseling a patient with a clinical T3 tumor or stage III disease, we could reference the current study and state that both surgery-based therapy and definitive CRT are good treatment options with the same oncologic efficacy. If the patient presented with an earlier or more advanced disease (T1–2, T4, stage II, or stage IV), the non-surgical approach tends to result in a significantly worse overall survival. Of note, our study has similar limitations as other series, such as a retrospective nature, small sample size, and no functional outcome.

**Table 4:** Summary of retrospective observational studies comparing surgery-based therapy versus definitive chemoradiotherapy.

Authors	Reference	Treatment period	No. of cases	% of stage III–IV	Surg vs. definitive CRT
Tassler et al.	23	1994–2014	137	Not available	*OS: Surg better (p = 0.02)
Thomas et al.	24	1994/01–2018/12	103	100.0%	*OS: Surg better (p = 0.049) *DFS: Surg better (p = 0.029)
Harris et al.	25	1999/01–2013/04	76	100.0%	OS: Surg better (p = 0.09) RFS: Surg better (p = 0.18)
Visini et al.	26	2003–2015	179	85.5%	Similar recurrence rate (57.5% vs. 46.8%)
Kim et al.	27	2002/01–2012/06	91	100.0%	Similar OS (56.6% vs. 58.6%) Similar DFS (52.7% vs. 51.0%)
Chung et al.	28	1997–2014	266	100.0%	Similar OS (p = 0.106), similar DFS (p = 0.194)
Jang et al.	29	1996–2014	332	75.9%	Similar OS (p = 0.991)
Chang et al.	30	1994/01–2004/05	395	93.9%	Similar OS, similar DSS
Chen et al.	31	2002/09–2013/09	257	100.0%	*OS: Surg better (53% vs. 32%, p < 0.001) *DFS: Surg better (48% vs. % 28%, p < 0.001)
Current study		2004/02–2012/12	167	90.4%	*OS (p < 0.0001), *PFS (p = 0.0002): Surg better Subgroup analysis: similar outcome only for stage T3 or overall stage III

Surg, surgery-based therapy; CRT, chemoradiotherapy; OS, overall survival; DFS, disease-free survival; RFS, recurrence-free survival; DSS, disease-specific survival; PFS, progression-free survival.

^*^Statistically significant difference.

## 5 Conclusion

In summary, there is no consensus on whether surgery-based therapy or definitive CRT should be the standard treatment approach to advanced HPC. Our new argument— definitive CRT approach—can achieve the same survival outcome as surgery-based therapy only for patients with T3 tumor or overall stage III disease, but needs a prospective phase III randomized trial with a large sample size to confirm.

## Data availability statement

The raw data supporting the conclusions of this article will be made available by the authors, without undue reservation.

## Ethics statement

This study was reviewed and approved by the Institutional Review Board of Taipei Veterans General Hospital. The committee waived the requirement of written informed consent for participation.

## Author contributions

P-YC formed the concept and designed the study. All authors contributed to data acquisition and quality control. T-YL and P-YC analyzed and interpreted the data. T-YL and P-YC wrote the manuscript. All authors edited the manuscript and approved the submitted version.
